# Supplementary material for: Topologically-guided continuous protein crystallization controls bacterial surface layer self-assembly
Source: Nat Commun. 2019 Jun 21;10:2731. doi: 10.1038/s41467-019-10650-x (PMC6588578; doi:10.1038/s41467-019-10650-x)
Supplement: Supplementary file 2 — Reporting Summary [file 41467_2019_10650_MOESM2_ESM.pdf]

nature research

Corresponding author(s): W.E. Moerner

Last updated by author(s): 5/9/2019

Reporting Summary

Nature Research wishes to improve the reproducibility of the work that we publish. This form provides structure for consistency and transparency in reporting. For further information on Nature Research policies, see Authors & Referees and the Editorial Policy Checklist.

Statistics

For all statistical analyses, confirm that the following items are present in the figure legend, table legend, main text, or Methods section.

n/a	Confirmed	
☐	☒	The exact sample size (*n*) for each experimental group/condition, given as a discrete number and unit of measurement
☐	☒	A statement on whether measurements were taken from distinct samples or whether the same sample was measured repeatedly
☒	☐	The statistical test(s) used AND whether they are one- or two-sided<br>*Only common tests should be described solely by name; describe more complex techniques in the Methods section.*
☒	☐	A description of all covariates tested
☒	☐	A description of any assumptions or corrections, such as tests of normality and adjustment for multiple comparisons
☐	☒	A full description of the statistical parameters including central tendency (e.g. means) or other basic estimates (e.g. regression coefficient) AND variation (e.g. standard deviation) or associated estimates of uncertainty (e.g. confidence intervals)
☒	☐	For null hypothesis testing, the test statistic (e.g. *F*, *t*, *r*) with confidence intervals, effect sizes, degrees of freedom and *P* value noted<br>*Give P values as exact values whenever suitable.*
☒	☐	For Bayesian analysis, information on the choice of priors and Markov chain Monte Carlo settings
☒	☐	For hierarchical and complex designs, identification of the appropriate level for tests and full reporting of outcomes
☒	☐	Estimates of effect sizes (e.g. Cohen's *d*, Pearson's *r*), indicating how they were calculated

Our web collection on statistics for biologists contains articles on many of the points above.

Software and code

Policy information about availability of computer code

Data collection	STED and confocal images were collected using custom software running on LabVIEW 2014. Tracking data was acquired using Andor SOLIS software (v. 4.28.30001.0).
Data analysis	Most image analysis was done using custom Matlab software, available upon reasonable request. 3D imaging calibration analysis was done using easyDHPSF. Surface reconstruction for Gaussian curvature analysis was performed using MeshLab.

For manuscripts utilizing custom algorithms or software that are central to the research but not yet described in published literature, software must be made available to editors/reviewers. We strongly encourage code deposition in a community repository (e.g. GitHub). See the Nature Research guidelines for submitting code & software for further information.

Data

Policy information about availability of data

All manuscripts must include a data availability statement. This statement should provide the following information, where applicable:

- Accession codes, unique identifiers, or web links for publicly available datasets
- A list of figures that have associated raw data
- A description of any restrictions on data availability

Supplementary figures (10) are provided with this manuscript, which include representative images from all data sets. Full imaging data sets are available from the corresponding author W.E.M. upon reasonable request.

Field-specific reporting

Please select the one below that is the best fit for your research. If you are not sure, read the appropriate sections before making your selection.

☒ Life sciences ☐ Behavioural & social sciences ☐ Ecological, evolutionary & environmental sciences

For a reference copy of the document with all sections, see nature.com/documents/nr-reporting-summary-flat.pdf

Life sciences study design

All studies must disclose on these points even when the disclosure is negative.

Sample size	No statistical methods were used to predetermine sample sizes. Sample sizes for S-layer localization experiments were >=50 cells (except Fig2d), which were sufficient to show trends.
Data exclusions	Out of focus images/cells were excluded from analysis. One cell was excluded from analysis in Fig 2d due to extreme cell length precluding accurate cell axis determination. Diffusion coefficient analysis excluded visibly bound tracks.
Replication	Every imaging experiment (except Fig 2e, 3e, and 3i) was replicated at least twice, yielding similar results.
Randomization	No randomization was required for this study.
Blinding	No blinding was necessary as image analysis was performed using identical code and parameters across independent variables.

Reporting for specific materials, systems and methods

We require information from authors about some types of materials, experimental systems and methods used in many studies. Here, indicate whether each material, system or method listed is relevant to your study. If you are not sure if a list item applies to your research, read the appropriate section before selecting a response.

Materials & experimental systems

n/a	Involved in the study	
☒	☐	Antibodies
☒	☐	Eukaryotic cell lines
☒	☐	Palaeontology
☐	☒	Animals and other organisms
☒	☐	Human research participants
☒	☐	Clinical data

Methods

n/a	Involved in the study	
☒	☐	ChIP-seq
☒	☐	Flow cytometry
☒	☐	MRI-based neuroimaging

Animals and other organisms

Policy information about studies involving animals; ARRIVE guidelines recommended for reporting animal research

Laboratory animals	Three strains of the alphaproteobacterium Caulobacter crescentus were used in this study.
Wild animals	n/a
Field-collected samples	n/a
Ethics oversight	Caulobacter crescentus is a BSL-1 bacterium and requires no ethical oversight for its use in a research environment.

Note that full information on the approval of the study protocol must also be provided in the manuscript.
